# An Unusual Case of Ventricular Tachycardia in a Young Patient Associated with Cannabis Use

**DOI:** 10.1155/2020/8813930

**Published:** 2020-12-01

**Authors:** Parth J. Sampat, Sana Riaz, Maneesh Bisen, Robert Carhart

**Affiliations:** ^1^Department of Medicine, SUNY Upstate Medical University, Syracuse, NY, USA; ^2^Department of Cardiology, SUNY Upstate Medical University, Syracuse, NY, USA

## Abstract

Marijuana has become the most widely used illicit drug in the United States. Approximately 43.5 million Americans aged 12 or above have reported the use of marijuana in the last year. The use of cannabinoids and its relationship with cardiac effects are not well known. Many types of arrhythmias have been noted with the use of cannabis products with atrial fibrillation being the most common arrhythmia associated with the use of cannabis. We present a case of a 36-year-old male who presented with pounding chest pain, dyspnea, and diaphoresis following marijuana use. He was found to be in ventricular tachycardia which responded to amiodarone. Workup done was negative for any structural disease, and cardiac catheterization was negative for coronary artery disease. He was ultimately discharged on metoprolol. In this report, we focus on how marijuana can be associated with many arrhythmias including ventricular tachycardia with focus on mechanisms by which it can occur. We believe a detailed social history with screening for cannabis use can identify more cases of arrhythmias that can be potentially associated with marijuana use.

## 1. Introduction

Marijuana has become the most used illicit substance in the United States [1]. Approximately 43.5 million Americans aged 12 or above have reported the use of marijuana in the last year [1]. Cannabinoids have various cardiovascular effects and involve the autonomic function in central and peripheral nervous systems and its direct effect on the myocardium [2]. There have been reports of multiple types of arrhythmias with the use of marijuana [3]. In one study, the prevalence of any type of arrhythmia with the use of marijuana has been found to be 4.66%, with atrial fibrillation having highest prevalence with the use of marijuana and ventricular tachycardia being associated 0.69% of the times [4]. In another paper, it was found that 2.7 percent of marijuana users developed arrhythmia [5]. There have also been reported cases of Brugada pattern on EKG with marijuana use [6–9]. A few cases of ventricular tachycardia and cardiac arrest have also been associated with marijuana use [10–14]. We present a case of a patient who presented with ventricular tachycardia in the setting of marijuana use and found to have no other cause of ventricular tachycardia.

## 2. Case Presentation

**Figure 1:**
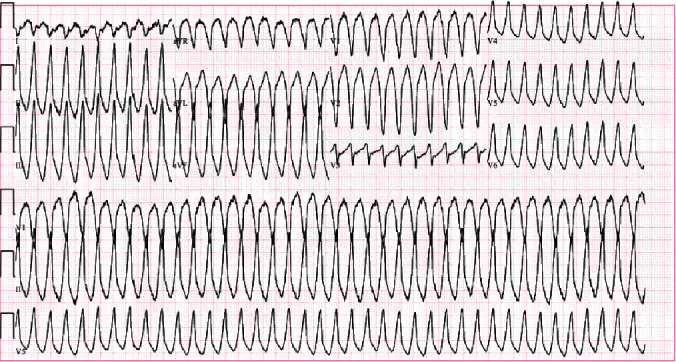
EKG showing right ventricular outflow tract ventricular tachycardia with left bundle branch pattern and inferior axis pattern.

**Figure 2:**
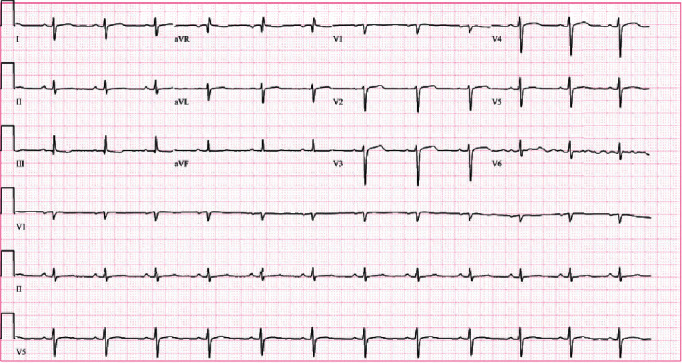
EKG showing normal sinus rhythm.

A 36-year-old male with past medical history most significant for morbid obesity, obstructive sleep apnea, and marijuana use disorder presented to the hospital with complaints of pounding chest pain and shortness of breath. He was sitting on his couch and smoking marijuana, after which he developed a pounding sensation in his chest, dyspnea, and diaphoresis that lasted approximately 30 seconds after which emergency medical service was called. His symptoms had resolved by the time he reached the hospital. After reaching the emergency room, his symptoms returned, and he was found to be in a monomorphic wide-complex tachycardia at a ventricular rate of 240 beats per minute (Figure 1) and was saturating at 89% at room air. His blood pressure was 133/84 mmHg and had a respiratory rate of 16. He was started on IV amiodarone 150 mg over 10 minutes and was continued on a drip with a rate on 1 mg/min, which resolved the arrhythmia after the initial bolus (Figure 2). He smokes 3 g marijuana daily. He denied the use of any drugs other than marijuana. He also denied any history of arrhythmia in the family.

An extensive workup was performed to find the cause of ventricular tachycardia. A urine toxicology report was significant only for cannabinoids in urine. Urine metanephrines and normetanephrines were within normal range. Serum magnesium was 1.7 mg/dL; potassium was 4.6 mmol/L at the time of admission. Troponin T levels were trended which remained negative for three occurrences. TSH was 0.903 IU/ml. Morning cortisol was 9.4 microgram/dL. Cardiac catheterization was performed to rule out ischemic causes of ventricular tachycardia, which showed normal coronaries. Echocardiogram obtained showed a left ventricular ejection fraction of 55-60% with normal valvular function, and no structural disease noted on echocardiogram. He was monitored on cardiac telemetry throughout his hospital course, and he was not found to have any further episodes of ventricular arrhythmias or ectopy.

With the consideration of ventricular tachycardia in a structurally normal heart and no coronary artery disease, he was started on metoprolol succinate 25 mg daily and was discharged with outpatient follow-up. He was provided education regarding marijuana cessation and was discussed.

He did undergo an electrophysiology study after discharge, which did not reveal any inducible ventricular or supraventricular tachycardia. A cardiac magnetic resonance imaging (MRI) was offered to the patient for further workup of arrhythmia; however, patient declined further workup. He has been continued on metoprolol succinate 25 mg and has remained asymptomatic since then.

## 3. Discussion

With workup largely negative, we believe that marijuana could have a potential role in the development of ventricular tachycardia in our patient.

Cannabis has over 400 active chemical entities with tetrahydrocannabinol (THC) being the most active chemical [15]. THC acts upon cannabinoid receptors CB_1_ and CB_2_ causing its effects on the heart. [2]

Mechanisms by which arrhythmias occur after use of marijuana remain unknown; however, multiple mechanisms have been proposed. The arrhythmogenic effects secondary to cannabis use may be related to its biphasic effects on the autonomic nervous system of the heart [16]. Lower doses cause sympathetic stimulation leading to tachycardia and increase in cardiac output, whereas higher doses cause parasympathetic stimulation [16]. Other likely mechanisms are increased myocardial oxygen demand, increased platelet activation, and coronary vasospasm leading to ischemic environment which can likely induce ischemia-induced arrhythmias even with the presence of nonobstructive coronary arteries [3]. Ischemia-induced arrhythmias can occur due to multiple mechanisms including damage to ion channels [17]. Decreased coronary flow has also been described in one care report associated with ventricular tachycardia and cannabis use [13].

With the increase in the use of marijuana, it is imperative to study the cardiovascular effects of marijuana. Even though our case does not show a causal effect of marijuana causing ventricular tachycardia, it is important to consider substance use when evaluating any type of arrhythmia. Screening of drug use by history and laboratory tests become essential in these situations.

## 4. Conclusion

Given various association of marijuana with cardiovascular effects in literature, we conclude that a detailed social history with screening for cannabis use by history of laboratory tests including urine toxicology studies can be helpful in determining the etiology of a new onset arrhythmia. More studies are required to prove association of marijuana with ventricular tachycardia.
